# Mechanical Mitral Valve Prosthesis Thrombosis: A Case Report

**DOI:** 10.15388/Amed.2025.32.1.13

**Published:** 2025-02-18

**Authors:** Viktorija Pareikaitė, Silvija Makrickaitė, Giedrė Bakšytė

**Affiliations:** 1 Faculty of Medicine, Medical Academy, Lithuanian University of Health Sciences; 2 Department of Cardiology, Lithuanian University of Health Sciences, Kaunas Clinics

**Keywords:** prosthetic valve thrombosis, thrombolysis, protezuotų vožtuvų trombozė, trombolizė

## Abstract

**Background:**

A rare but serious complication of heart valve replacement, prosthetic valve thrombosis carries significant risks of morbidity and mortality. Effective management depends on prompt diagnosis and the appropriate treatment, often involving fibrinolytic agents. Protocols using slower infusion rates and lower doses of these agents have led to improved therapy outcomes.

**Clinical case:**

We report a case of a 56-year-old man admitted to the Lithuanian University of Health Sciences Kaunas Clinics due to mechanical mitral valve prosthesis thrombosis complicated by a respiratory failure and atrial fibrillation. The patient was treated with ultraslow thrombolysis with alteplase. The function of the mechanical valve prosthesis became normal, and the patient was discharged from the hospital.

**Discussion and Conclusions:**

Managing prosthetic valve thrombosis is challenging due to overlapping clinical features with other diagnoses and the lack of consensus on the treatment methods. Slow-infusion, low-dose thrombolytic therapy with alteplase can be a life-saving intervention with a high success rate.

## Abstract (it)

**Įvadas:**

**:** Protezuoto širdies vožtuvo trombozė yra reta, tačiau labai rimta komplikacija, kelianti didelę sergamumo ir mirtingumo riziką. Veiksmingas gydymas priklauso nuo greito diagnozės nustatymo ir tinkamo gydymo, dažnai pasirenkant fibrinolizinius vaistus. Skiriant šiuos vaistus lėta ar ultralėta infuzija ir mažomis dozėmis, būna geresni gydymo rezultatai.

**Klinikinis atvejis:**

**:** Pristatome 56 metų vyro, hospitalizuoto į Lietuvos sveikatos mokslų universiteto Kauno klinikas dėl mechaninio mitralinio vožtuvo protezo trombozės, komplikuotos kvėpavimo nepakankamumu ir prieširdžių virpėjimu, klinikinį atvejį. Pacientas buvo gydomas ultralėta trombolize alteplaze. Mechaninio mitralinio vožtuvo protezo funkcija tapo normali ir pacientas buvo išrašytas iš ligoninės.

**Diskusija ir išvados:**

**:** Protezuoto širdies vožtuvo trombozė yra sudėtinga patologija dėl nespecifinių klinikinių požymių bei bendro sutarimo dėl gydymo metodų trūkumo. Ultralėta trombolizė mažomis alteplazės dozėmis gali būti gyvybę gelbstinti intervencija, lemianti geras prognozes.

## Introduction

*Prosthetic Valve Thrombosis* (PVT) is a rare but serious complication following a heart valve replacement, often linked to significant morbidity and mortality [1]. Timely diagnosis and suitable treatment are crucial for the effective management of the issue. Treatment options typically include surgical intervention, fibrinolysis, and heparin therapy, depending on the patient’s clinical condition. The choice of the most effective treatment is largely influenced by the status of the valve. Factors such as the presence of valvular obstruction and the thrombus location (left or right side) can affect the treatment strategy [2].

Fibrinolytic agents are one of the treatment options for PVT. There has been a notable improvement in the outcomes of fibrinolytic therapy following the implementation of protocols involving slow or ultraslow infusion rates and lower doses of fibrinolytic agents. When compared to previous protocols which used high doses and rapid infusions, the new methods demonstrated a success rate of up to 90% versus 75%, a thromboembolic (TE) rate of less than 2% compared to 13%, and a major bleeding rate of under 2% compared to 6% [3].

Here, we shall report a clinical case of PVT diagnosed on a mechanical mitral valve and the successful treatment with low-dose, ultraslow-infusion alteplase without complications.

## Clinical case

A 56-year-old man presented with intense shortness of breath during lunch, which only decreased when he sat down. Earlier that same day, he had been working in a garage, painting a car with aerosol paints. The patient reported that he had inhaled aerosol paints, and, after lunch, shortness of breath developed. He called an ambulance and was transported to a local hospital.

The patient’s medical history was obtained. In 2012, he was diagnosed with rheumatic heart disease, which resulted in mitral valve stenosis and third-degree mitral valve prolapse. A *Medtronic* 29 mm mitral valve prosthesis was subsequently implanted. The patient also had arterial hypertension, heart failure, and dyslipidemia, and also had a history of paroxysmal atrial fibrillation. He was taking warfarin 5 mg, metoprolol 47.5 mg, and perindopril 5 mg daily.

Laboratory tests showed a significant increase in NT-proBNP (6223 pg/ml), a moderate increase in troponin I (with negative dynamics, measuring 35.8 and 51.1 ng/l) and a slight increase in d-dimers (3.81 mg/l). Additionally, therapeutic hypocoagulation was not achieved, as the INR was 0.97. According to the patient, he had been taking warfarin continuously. A chest *Computed Tomography* (CT) scan was performed for the differential diagnosis of dyspnea. The findings were insufficient for diagnosing pulmonary embolism. However, the CT indicated signs of pulmonary hypertension. The patient received intravenous furosemide 20 mg. For further investigation and treatment, he was transported to the Emergency Department of Lithuanian University of Health Sciences Kaunas Clinics.

The patient’s physical examination at the Emergency Department revealed a blood pressure of 109/77 mmHg, a heart rate of 98 beats/min, a respiratory rate of 18 breaths/min, and vesicular breath sounds with audible moist rales in the lower lung fields. His oxygen saturation was 98% with 10 L/min of oxygen via a mask, and there were no signs of edema. An EKG showed tachysystolic atrial fibrillation.

Laboratory tests showed normal hemoglobin (139 g/L), elevated leukocytes (12 x 10^9/L), elevated neutrophils (9.2 x 10^9/L), a decreased platelet count (127 x 10^9/L), elevated CRP (44.1 mg/L), normal creatinine (81 µmol/L), normal potassium and sodium levels, and slightly decreased magnesium (0.72 mmol/L). Therapeutic hypocoagulation was not achieved (INR 1.1, APTT 32.6 s), and elevated troponin I levels were noted with positive dynamics (70.4 and 65.8 ng/L).

A chest X-ray performed at the Emergency Department showed venous stasis and infiltrative changes in the lower part of the right lung. Suspecting mitral valve dysfunction, a *Transthoracic Echocardiogram* (TTE) was performed, which revealed a decreased systolic function of the left ventricle due to dyssynchronous contraction and tachysystolic atrial fibrillation. The left atrium was significantly dilated, and the blood flow through the *Mitral Valve* (MV) prosthesis significantly increased (from 1.4 to 2.78 m/s). The TTE also showed a dilated right atrium, a decreased longitudinal right ventricular function, and II–III° tricuspid valve regurgitation.

In the Emergency Department, the patient received intravenous furosemide 100 mg, fraxiparine 0.6 mL subcutaneously, intravenous ketorolac 100 mg, and metoprolol 50 mg. Due to the suspicion of a mitral valve dysfunction and the potential exacerbation of heart failure, he was transferred from the Emergency Department to the cardiology intensive care unit.

A *Transesophageal Echocardiogram* (TEE) was performed, which showed mitral valve prosthesis dysfunction, with both valve leaflets moving with a reduced amplitude. Thrombosis of the mitral valve prosthesis was suspected, and the left atrium was found to be dilated. A decrease in the inflow and outflow velocities in the left atrial appendage was also observed.

At the cardiology intensive care unit, the patient received an intravenous bolus of heparin (5000 units) and continuous heparin administration via an automatic syringe pump. He was also given intravenous metoclopramide (10 mg) and diazepam (10 mg) before the TEE, as well as intravenous digoxin (0.25 mg), metoprolol (47.5 mg), perindopril (4 mg), lansoprazole (30 mg), torasemide (10 mg), and spironolactone (25 mg).

During the course of treatment, the patient’s general condition worsened, and progressive respiratory insufficiency was noted despite maintaining therapeutic hypocoagulation with optimal doses of heparin. Consequently, it was decided to initiate ultraslow thrombolysis with alteplase. Alteplase was infused at a rate of 2 mg per hour for 25 hours, totaling 50 mg.

After thrombolytic treatment, significant improvement in the patient’s clinical condition was observed, and the sinus rhythm was restored. A repeat TTE revealed a dilated left atrium. The blood flow through the MV prosthesis significantly decreased (from 2.78 to 2 m/s), and stenosis was assessed as moderate. Furthermore, the TTE showed that the right atrium was no longer dilated, and that the longitudinal right ventricular function was good. I–II° tricuspid valve regurgitation was still present. Warfarin was initiated.

Additionally, intravenous cefuroxime (1.5 g three times a day) was started on the second day of treatment at the cardiology intensive care unit due to increased inflammatory markers (CRP 229.3 mg/L), the patient’s fever, and signs of infiltration observed on the chest X-ray. The antibiotic therapy was continued throughout the hospital stay, and, by the time of discharge, the CRP value had dropped to 5 mg/L.

After six days of treatment at the cardiology intensive care unit, the patient was transferred to the Department of Heart Failure. At this department, he received metoprolol (23.75 mg twice daily), lansoprazole (30 mg), torasemide (10 mg), and spironolactone (25 mg). Heparin administration continued via an automatic syringe pump according to the APTT values. After achieving therapeutic hypocoagulation with warfarin, heparin administration was discontinued. The patient received warfarin doses of 10 to 15 mg, adjusted according to INR.

On the ninth day of hospitalization, TTE was repeated, showing that the blood flow through the MV prosthesis decreased from 2 m/s to 1.77 m/s

The patient’s overall condition improved, and he was discharged from the hospital. He will continue treatment with warfarin (15 mg, target INR 2.5–3.5 due to mechanical mitral valve prosthesis), metoprolol (47.5 mg), and perindopril (5 mg). The target blood pressure is 120–129/80 mmHg. The patient is advised to have weekly ambulatory INR assessments, and, once stabilized, at least monthly checks.

## Discussion

Prosthetic valve thrombosis is a serious condition where a blood clot forms on or around a mechanical heart valve, obstructing the blood flow. Although mechanical prosthetic valves are more durable than bioprosthetic ones, they carry a higher risk of clot formation and require a lifelong anticoagulant therapy. The occurrence of PVT is rare, affecting 0.1 to 5.7% of patients annually, but it poses a significant threat to life [4]. It is also of importance to note that mitral valves have a higher risk of thrombosis than aortic valves due to the slower blood flow in the mitral position [5].

Various factors contribute to the development of prosthetic valve thrombosis, resulting in obstruction. The most common cause is an insufficient anticoagulation therapy, followed by conditions promoting clot formation, such as atrial fibrillation and ventricular dysfunction [6].

PVT can present with different clinical scenarios based on the size of the thrombus and its impact on the prosthetic valve function. The symptom severity largely depends on the extent of valve obstruction caused by the thrombosis. The size, location, and whether the thrombus develops suddenly or gradually all influence the intensity of symptoms. Clinical manifestations can vary from severe conditions such as acute heart failure, including acute pulmonary edema or cardiogenic shock, to milder symptoms such as reduced exercise tolerance, fatigue, and shortness of breath (dyspnea) [7].

There are a variety of imaging modalities that are used to identify the etiology, location, severity, and hemodynamic changes associated with PVT. The key findings include increased valvular gradients, a restricted valve mobility, regurgitation, and direct visualization of the thrombus. According to the ESC and ACC/AHA guidelines, patients with suspected mechanical prosthetic valve thrombosis should undergo urgent evaluation using *Transthoracic Echocardiography* (TTE), *Transesophageal Echocardiography* (TEE), cinefluoroscopy, and/or multidetector computed tomography (MDCT) [2,8]. Assessing prosthetic valve dysfunction via TTE is more challenging than with the native valves. Due to the reverberations of the disc, the atrial side of the mitral prosthesis and the posterior side of the aortic prosthesis are difficult to visualize [5]. Nevertheless, TTE remains the first-line imaging modality for evaluating dysfunctional prosthetic valves, with a sensitivity and specificity of approximately 75% and 57%, respectively. Initially, thrombosis on the prosthesis is suspected due to high prosthesis gradients and velocities [9]. TEE is considered the gold standard for diagnosing PVT, particularly in cases where thrombosis obstructs the mechanical valve. Its proximity to the left atrium allows for clear visualization of the atrial surface of mitral prostheses [9,10]. TEE excels at identifying the thrombus size, mobility, and extent, and can distinguish between obstructive and non-obstructive thrombi, which is crucial for determining the treatment options [11]. Cinefluoroscopy, which is a noninvasive method, is useful for assessing the motion of valve leaflets, especially when TTE images are inconclusive. However, while cinefluoroscopy provides the most accurate assessment of mechanical leaflet motion, it cannot detect soft tissues like thrombus or pannus [12]. Although TEE is the gold standard, MDCT is increasingly used, particularly when the obstruction diagnosis is unclear, as it is valuable for differentiating between thrombus and pannus, as emphasized in the ESC guidelines [8, 13].

In our case, the symptoms and signs of heart failure, including acute dyspnea, raised levels of NTproBNP, with a history of a prosthetic valve raised the suspicion of prosthetic dysfunction. In addition, the recommended therapeutic anticoagulation was not achieved, even though the patient claimed he had been taking warfarin daily. Findings from TTE and TEE, including an elevated pressure gradient across the prosthesis along with an elevated velocity and a reduced mitral valve leaflet motion raised the concern for a prosthetic valve obstruction. Our primary differential diagnoses were pulmonary embolism and myocardial infarction, but these were ruled out through laboratory tests and a chest CT scan.

Prosthetic valve dysfunction is a life-threatening condition requiring urgent treatment. For patients with thrombosis of a left-sided mechanical prosthetic heart valve, the ACC/AHA guidelines recommend either a surgery or slow-infusion, a low-dose fibrinolytic therapy (25 mg of tissue-type plasminogen activator administered over 6 to 24 hours without a bolus) as Class I interventions [2]. The ESC guidelines, however, prioritize a surgery and suggest a standard-dose fibrinolytic therapy (10 mg bolus of recombinant tissue plasminogen activator followed by 90 mg over 90 minutes with unfractionated heparin) as a Class IIa recommendation when a surgery poses high risk or is not available [8]. Determining the optimal treatment can be challenging due to the lack of randomized controlled trials. While anticoagulation with heparin should begin immediately after diagnosing prosthetic valve thrombosis, definitive treatment – either a fibrinolytic therapy or a surgery – must be considered. Clinical trials indicate that a low-dose, slow-infusion tissue plasminogen activator therapy is highly effective for PVT. For instance, the Ultraslow PROMETE (*PROsthetic MEchanical valve Thrombosis and the prEdictors of outcomE*) trial reported a 90% success rate with an ultraslow alteplase regimen, along with a low mortality rate (0.8%) and the overall complication rate (6.7%) [14].

Once PVT was suspected, our patient was placed on the heparin therapy. However, as the patient’s condition deteriorated, the decision was made to initiate the fibrinolytic therapy. Following the administration of alteplase at 2 mg per hour over 25 hours, totaling 50 mg, the patient’s symptoms improved significantly, with a restored normal valve leaflet motion and blood flow through the valve. Since the patient did not achieve the recommended therapeutic anticoagulation, his INR target was not adjusted, and values in the range of 2.5 to 3.5 were advised.

## Conclusions

Management of prosthetic valve thrombosis poses significant challenges, primarily due to the similarities in clinical features across various diagnoses and the lack of randomized controlled trials comparing different treatment methods. It is essential to maintain a high level of suspicion when indicative signs and symptoms are present, together with multimodal imaging, which plays a crucial role in assessing and diagnosing prosthetic valve dysfunction. Slow-infusion, low-dose thrombolytic therapy can be a life-saving intervention and an important strategy to prevent the necessity for high-risk repeated surgeries.
